# Predicting the hyperelastic properties of alginate-gelatin hydrogels and 3D bioprinted mesostructures

**DOI:** 10.1038/s41598-023-48711-3

**Published:** 2023-12-09

**Authors:** Anahita Ahmadi Soufivand, Silvia Budday

**Affiliations:** Institute of Continuum Mechanics and Biomechanics, Department of Mechanical Engineering, Friedrich-Alexander-Universität Erlangen-Nürnberg, 91058 Erlangen, Germany

**Keywords:** Mechanical engineering, Gels and hydrogels, Biomedical engineering, Computational science

## Abstract

Additive manufacturing has been widely used in tissue engineering, as 3D bioprinting enables fabricating geometrically complicated replacements for different tissues and organs. It is vital that the replacement mimics the specific properties of native tissue and bears the mechanical loading under its physiological conditions. Computational simulations can help predict and tune the mechanical properties of the printed construct—even before fabrication. In this study, we use the finite element (FE) method to predict the mechanical properties of different hydrogel mesostructures fabricated through various print patterns and validate our results through corresponding experiments. We first quantify the mechanical properties of alginate-gelatin hydrogels used as matrix material through an inverse approach using an FE model and cyclic compression-tension experimental data. Our results show that the fabrication process can significantly affect the material properties so that particular caution needs to be paid when calibrating FE models. We validate our optimized FE model using experimental data and show that it can predict the mechanical properties of different mesostructures, especially under compressive loading. The validated model enables us to tune the mechanical properties of different printed structures before their actual fabrication. The presented methodology can be analogously extended for cell bioprinting applications, other materials, and loading conditions. It can help save time, material, and cost for biofabrication applications in the future.

## Introduction

3D bioprinting is an emerging technology to fabricate complicated biological constructs layer by layer^1–5^. The constructs’ mechanical properties should be similar to those of the target tissue to properly bear the physiological loading and function^6,7^. However, the mechanical properties of tissues may differ, and it is therefore necessary to carefully control the design and fabrication process to print tissue mimetic replacements. The mechanical properties of the matrix material and the printing pattern are two influential parameters for tuning the properties of the final construct^8–12^. Different materials have previously been used in the biofabrication field, such as thermoplastics^13,14^, ceramics^15^, and hydrogels^16–18^. However, only hydrogels can be used for cell printing in soft and hard tissue engineering applications. Alginate-gelatin (AG) hydrogels have proven expedient as they are easy to prepare and use and provide a cell-friendly environment^18–20^. Moreover, using an appropriate fabrication method makes it possible to bioprint multilayer mesostructures with AG hydrogels^21^.

The tissue-engineered constructs must be porous to deliver oxygen and nutrition to the cells^22–24^. Tuning the mechanical properties of tissue replacements can be achieved by changing the porosity through geometrical parameters of pore size, layer height, and filament diameter^25,26^. However, the experimental approaches are time-consuming. Therefore, computational simulations are a valuable alternative approach to tune the mechanical properties of the construct with various mesostructures without any fabrication—saving both time and cost. The finite element (FE) method is a powerful tool for simulating the behavior of a system, which has been widely utilized in different fields^27,28^. In tissue engineering, FE approaches showed great potential, especially in the area of 3D printing^12,29,30^.

In FE simulations, the material model and corresponding parameters are a determinant factor for the predictions' accuracy^11^. Therefore, it is necessary to experimentally characterize the hydrogel properties to be able to identify an appropriate model and parameters for the simulations. For this purpose, the FE approach can be used directly^12^ or inversely^31^ to extract material parameters from mechanical testing data. A direct procedure can be utilized when the testing setup ensures homogeneous deformations. However, this is difficult when testing soft materials. Therefore, an inverse approach should be used to obtain accurate material parameters^32,33^. Herein, the testing setup is modeled in the FE software environment and realistic loading conditions are applied. Then, the numerical prediction is compared to the actual experimental value, and the material model and parameters are optimized to minimize their difference.

Among different material models previously used for hydrogel materials^16,34^, we chose the hyperelastic Ogden model. This model has proved appropriate for hydrogel material modeling as well as for the mechanical characterization of bioprinted constructs and native soft tissues^16,35–38^. Especially when mechanical testing involves large deformations, it is necessary to use nonlinear material models. We focus only on time-independent properties and thus neglect viscoelastic effects^39,40^. To the best of our knowledge, few research has been done on the hyperelastic material characterization of hydrogels in the 3D bioprinting field. One study fit compressive test data of bioprinted tissue analytically to different hyperelastic models (Exponential function, power function, and Ogden model)^38^. However, an inverse identification of parameters based on both tension and compression data simultaneously and the proper validation of simulation models remains unexplored.

**Figure 1:**
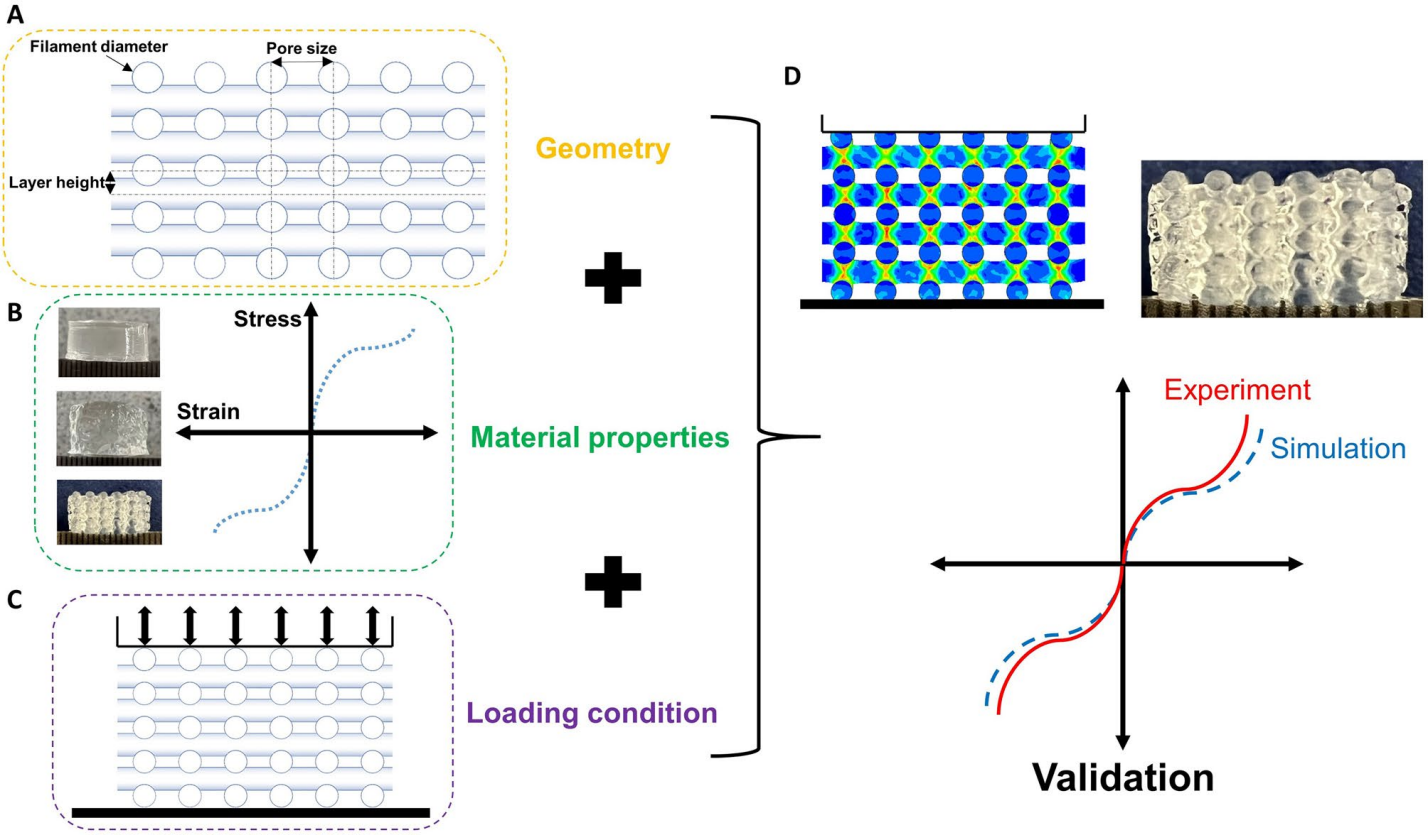
Schematic overview of the required steps for developing and validating a finite element (FE) model to predict the mechanical properties of 3D bioprinted tissue constructs. (**A**) Filament diameter, pore size and layer height are defined in the geometry. (**B**) The material model and parameters are identified and implemented in the model. (**C**) The actual experimental loading is simulated and (**D**) the simulation results are compared to the experimental data for validation.

This study aims to predict the nonlinear mechanical behavior of multilayer bioprinted hydrogel mesostructures through finite element simulations. We first characterize the AG hydrogel material through experiments and identify material parameters for the Ogden model using an inverse FE approach and least square optimization. Subsequently, we design different mesostructures by changing pore size, layer height, and filament diameter. We then simulate the behavior of these printed structures under large-strain compression-tension loadings. We predict the mesostructures' mechanical properties and validate our simulation results using associated experimental data. Finally, we use the validated model to assess the mechanical behavior of other mesostructures with various printing patterns and introduce a wide range of mechanical properties for soft tissue engineering applications (Fig. 1).

## Materials and methods

### Sample preparation

We used the experimental data of our previous study on multilayer bioprinting of AG hydrogel^21^. Briefly, alginate (type PH163) was purchased from Vivapharm, JRS PHARMA GmbH & Co. KG, and gelatin (type A, 300 bloom derived from porcine skin) was purchased from Sigma Aldrich (Germany). We prepared the AG bioink with 2% (w/v) and 5% (w/v) ratios of alginate and gelatin, respectively. Then, the printed samples were printed based on a cylindrical model with dimensions similar to molded samples and 100% infill. In addition, for preparing macroporous samples, after designing and preparing their printing files, we set the printing parameters, e.g., nozzle diameter and layer height on the BioX bioprinter (BICO, Sweden) interface. Then, we fabricated the final samples by printing larger macroporous constructs with the height of 4 mm and extracting cylindrical samples by using an 8 mm surgical punch. Finally, we placed all the samples in 0.1 M CaCl_2_ crosslinking solution for about 10 min and washed them with Hanks' Balanced Salt Solution (HBSS) purchased from ThermoFisher, Invitrogen, Germany.

To name the mesostructure type, we used the DxPyHz format, where x, y, and z stand for filament diameter, pore size, and percentage of layer height to filament diameter, respectively. For example, D6P6H75 is a mesostructure with a filament diameter and pore size of 600 µm, and the layer height is 75% of the filament diameter, which equals 450 µm. The exact final geometrical parameters of prepared samples are presented in Table 1.

**Table 1:** Averaged geometrical specifications with standard deviation of different molded, printed and porous samples used in this study.

Sample type	Diameter (mm) ± SD	Height (mm) ± SD
Molded	8.00 ± 0.07	3.76 ± 0.09
Printed	7.10 ± 0.19	5.29 ± 0.10
D4P6H75%	7.62 ± 0.06	3.49 ± 0.16
D5P6H75%	7.75 ± 0.26	3.70 ± 0.16
D6P6H75%	7.43 ± 0.13	3.70 ± 0.24
D6P6H67%	7.48 ± 0.07	3.97 ± 0.13
D6P6H83%	7.70 ± 0.31	3.52 ± 0.06
D6P9H75%	7.52 ± 0.25	3.65 ± 0.14
D6P12H75%	7.64 ± 0.32	3.51 ± 0.06

### Mechanical measurements

**Figure 2:**
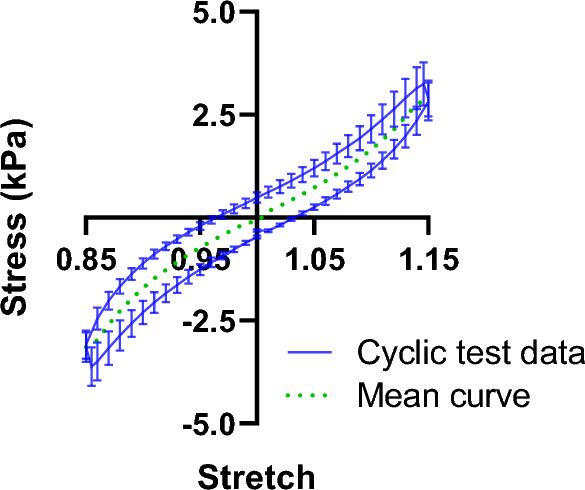
Extracting the mean curve from the cyclic compression-tension testing data from^21^ for use in hyperelastic FE simulation and validation. The curves are exemplary shown for the third cycle of testing the D6P6H75 sample.

For mechanical testing, we performed cyclic compression-tension tests using a Discovery HR-3 rheometer (TA instruments, New Castle, Delaware, USA) equipped with an 8 mm diameter parallel geometry, as described in detail in Ref.^21^. Briefly, we glued an 8 mm circular piece of fine sandpaper to the top (upper loading surface) geometry and attached the samples to it using an instant adhesive (super glue gel, Pattex). Then, we lowered the top geometry to glue the sample to the bottom heat plate (lower loading surface) with a preload < 0.1 N. Next, we immersed the sample in an HBSS bath at 37 °C to mimic the in vivo conditions to avoid sample dehydration during testing. Then, we performed cyclic compression-tension tests with three loading cycles, from 0.85 to 1.15 stretches at the loading rate of 40 μm/s. In this study, we used the mean curve of the third cycle for our FE modeling and validation (Fig. 2), as we limited ourselves to a time-independent Ogden hyperelastic model to characterize the hydrogel material.

### Ogden material model

We assumed the hydrogel to be incompressible due to its high water content and limited ourselves to the one-term Ogden model^35^. Therefore, the hydrogel can be characterized by two material parameters according to the strain energy function

1 $${U}_{Ogden}=\frac{2\mu }{{\alpha }^{2}}\left({\lambda }_{1}^{\alpha }+{\lambda }_{2}^{\alpha }+{\lambda }_{3}^{\alpha }-3\right),$$

where $\mu$ is the shear modulus, $\alpha$ the nonlinearity parameter, and ${\lambda }_{i}$ are principal stretches. Abaqus uses this equation to simulate the hyperelasticity by two material parameters using one-term Ogden model.

We checked the Drucker stability condition after material parameters identification to evaluate whether they were physically possible^31^.

### Material parameter identification through inverse FE simulations

As the experiments led to inhomogeneous deformation states during testing, it was important to use an inverse approach based on FE simulations accounting for the actual experimental boundary conditions to ensure obtaining accurate material parameters. Generally, a model with initial arbitrary material parameters is simulated, and the difference between results and experimental data is calculated. Then, this difference is minimized by repeatedly changing material parameters and analyzing the model in the optimization step. Here, we used the experimental data from our previous study^21^ to identify the material properties of the alginate 2% (w/v)-gelatin 5% (w/v) hydrogels. We considered three groups, samples that were molded, printed with 100% filling, as well as porous samples with a filament diameter and pore size of 600 µm and a layer height of 75% of the filament diameter.

**Figure 3:**
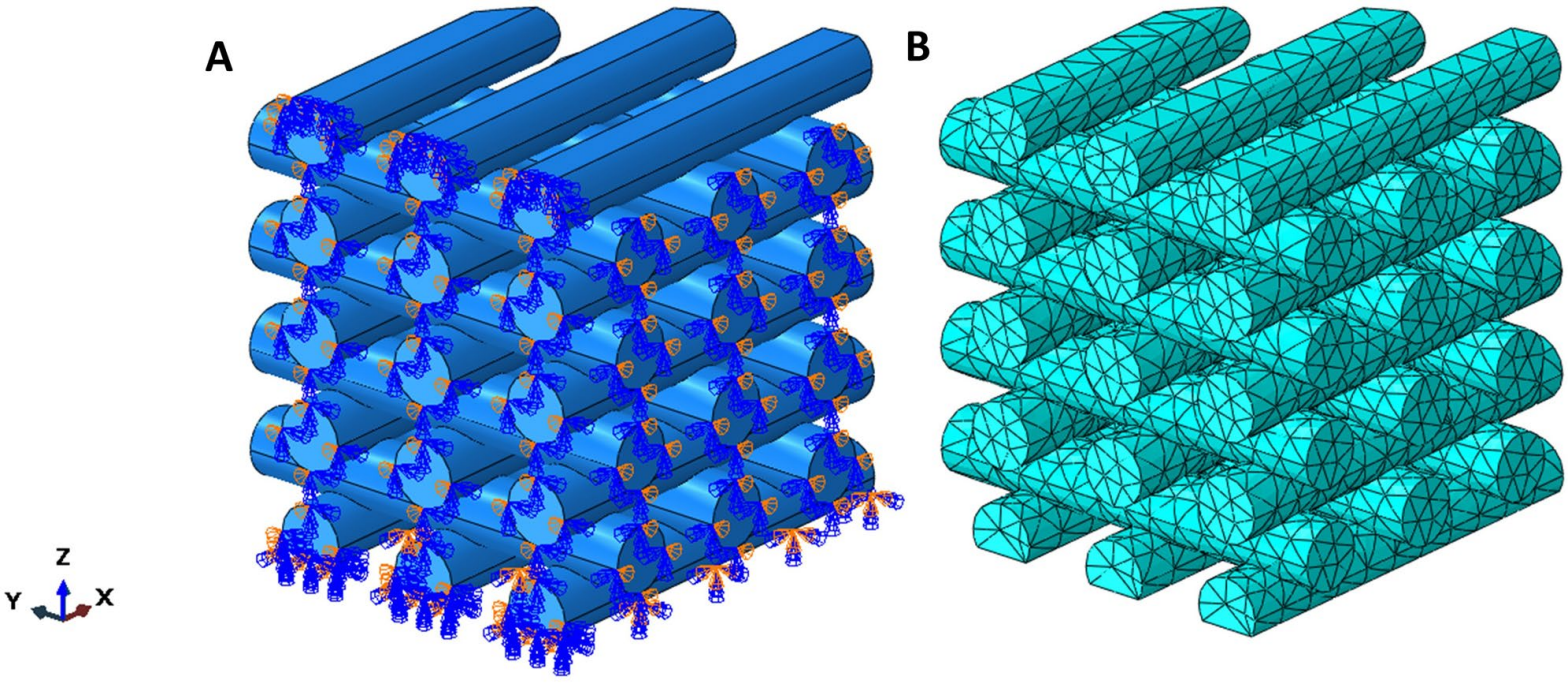
Boundary conditions of the symmetric quarter geometry (**A**) and the generated quadratic tetrahedral (C3D10H) elements for FEM analysis (**B**).

We generated axisymmetric FE models for molded and printed groups and a symmetric quarter model for the porous sample using the ABAQUS/Standard^®^ 2021 (Simulia, DassaultSystèmes) software to save computational costs. Figure 3A exemplary shows the model with symmetric boundary conditions in the x and y directions. We cut the upper layer (equal to 0.05 mm) to exert displacement loading in the z direction and to resemble that the soft strands do in reality not have a line contact with the upper specimen holder at the top surface. We fix other translational and rotational degrees of freedom to resemble the glued condition during the experiments. In addition, to simulate the first layer attachment to the bottom plate at the start of printing, this layer was cut (equal to 25% of filament diameter). Finally, the bottom surface was fixed in all directions to imitate that the sample was glued to the bottom plate during mechanical testing. Due to the complex geometry, we chose tetrahedral elements, hybrid quadratic 3D stress (C3D10H) elements (Fig. 3B) and performed a mesh sensitivity analysis to ensure independent results from the mesh size. To achieve accurate results for the curved edges and surfaces in the model, we used quadratic elements.

**Figure 4:**
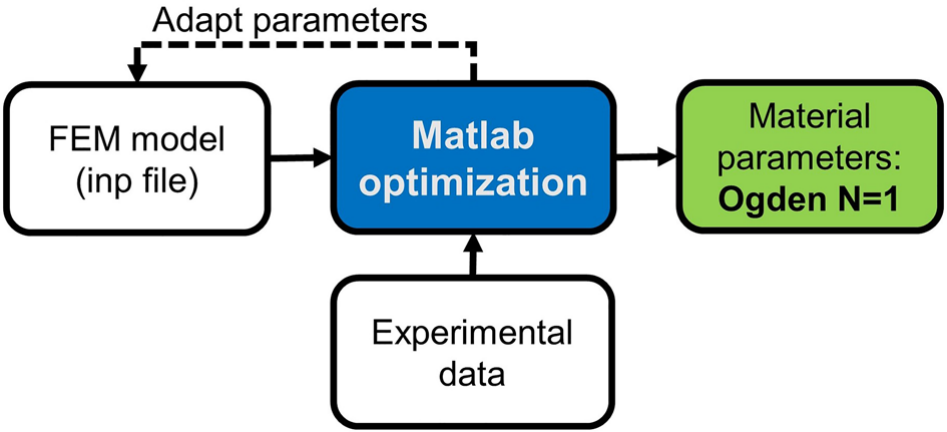
Parameter identification process steps to find the material parameters of AG hydrogel using the one-term Ogden hyperelastic material model. After generating the input file from the FE model, the material parameters were adapted in Matlab until reaching the numerical results close enough to the experimental data.

The compression-tension loading was simulated by performing two static steps with nonlinear geometric effects: head displacement to 0.85 initial height and, subsequently, from 0.85 to 1.15. We also defined arbitrary values for parameters of the one-term Ogden model. After generating an input file (.inp) from the model, this file was analyzed, and the results were obtained. Then, these numerical results were compared with the experimental data to calculate the difference. If it was not small enough, the material parameters were changed in the input file for a new finite element simulation, and this process was repeated until reaching a threshold of 0.0002. For this optimization procedure visualized in Fig. 4, we used the fmincon function in Matlab 2020b (MathWorks) to find the best material parameters to fit the experimental data. We used ABAQUS/Standard® 2021 (Simulia, DassaultSystèmes) for the FEM analysis.

### FE simulations

We designed seven mesostructures similar to what we had printed in our previous study^21^ with different pore sizes, layer heights, and filament diameters using Solidworks 2019 (Dassault Systemes) (Fig. 3). To reduce the computational cost, we imported the 3D models in the symmetric quarter geometry to ABAQUS.

Finally, we used the three identified sets of material parameters (molded, printed, and porous) to simulate the behavior of the various mesostructures printed and tested in our previous study^21^. The boundary and loading conditions were similar to those used for the inverse parameter identification because our experimental setup was identical for all samples.

After mesh generation with C3D10H elements, we again performed a mesh sensitivity analysis. Finally, we plotted the extracted nominal stress from the numerical predictions versus the experimental data for validation.

**Figure 5:**
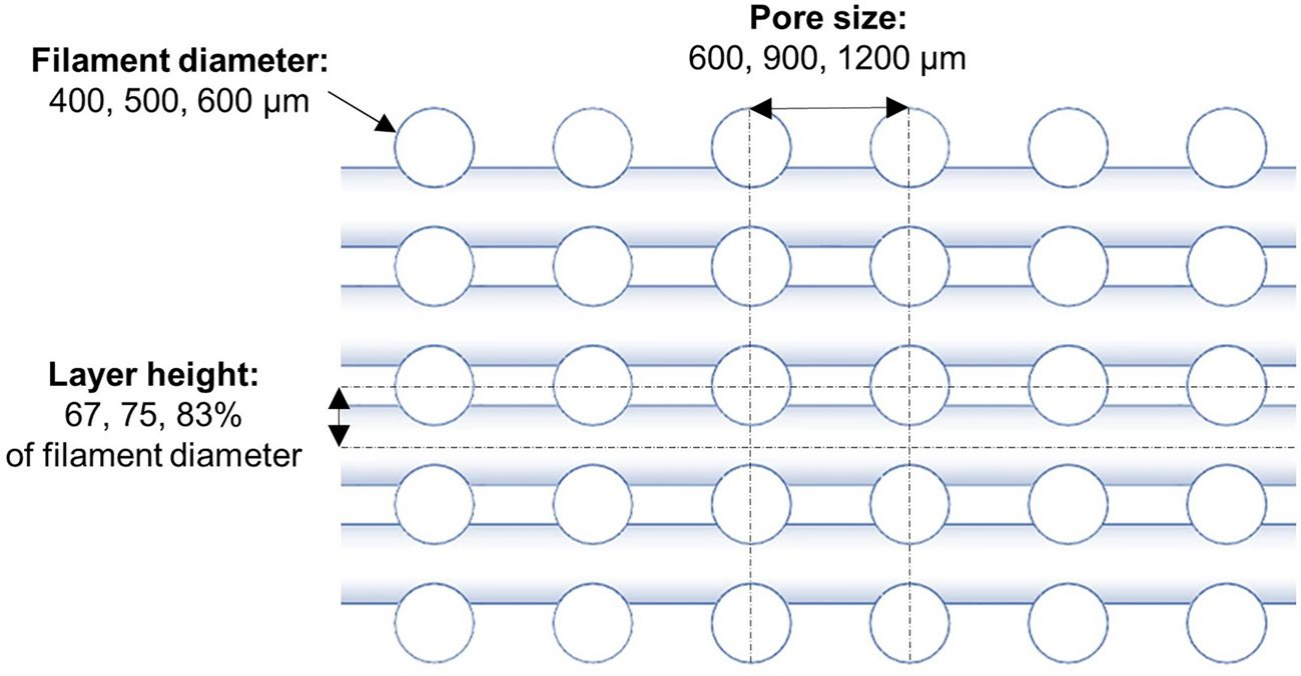
The geometrical parameters of the designed mesostructures. The filament diameters are 400, 500 and 600 µm; the pore sizes are 600, 900 and 1200 µm and the layer heights are 67, 75 and 83% of filament diameter.

In a last step, we used the validated FE model to predict the behavior of twenty-seven possible mesostructures using three chosen values for each geometrical parameter (Fig. 5).

## Results

### Material parameters

**Figure 6:**
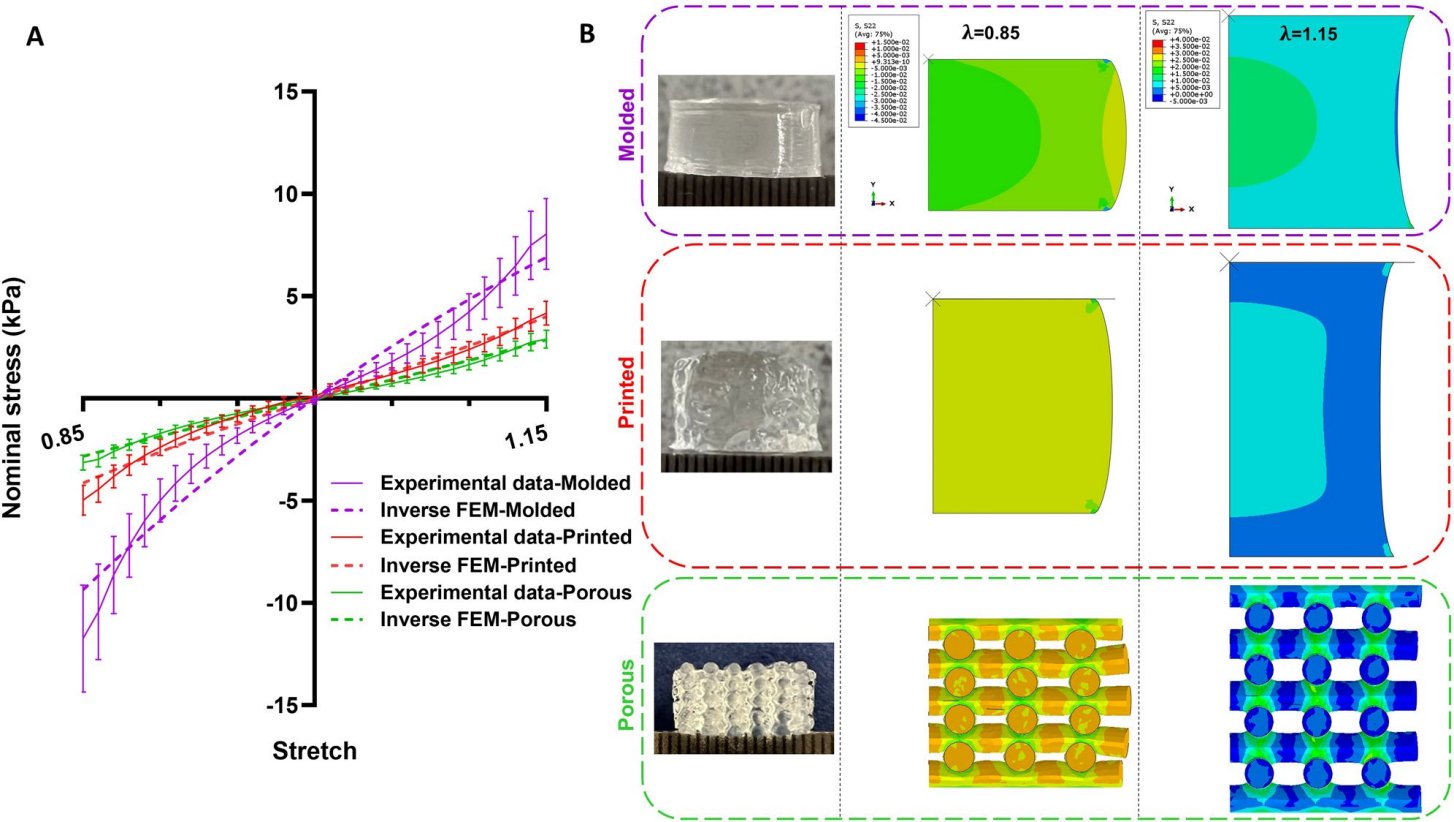
Comparison of experimental data and finite element model predictions for molded, printed, and porous samples (**A**) with corresponding actual sample shape and cross-sectional stress distribution at the stretches of 0.85 and 1.15 (**B**).

We identified material parameters using an inverse approach through finite element (FE) simulations, as described in detail in Section "Material parameter identification through inverse FE simulations". We determined three different sets of parameters based on the average experimental data for molded, printed, and D6P6H75 porous samples. Figure 6A demonstrates that the Ogden model with one term, N = 1, fits the experimental data well. Figure 6B shows the corresponding samples as well as the simulated cross-sectional stress distributions at maximum compression (λ = 0.85) and tension (λ = 1.15). The numerically predicted stress levels are higher in the molded samples compared to printed samples, in good agreement with experimental data. The stress distribution is entirely different in the porous sample with higher stress levels at layers junctions. In contrast, we see low amounts of stress in non-bonding regions as well as tensile stresses (positive stress values) under compression and compressive stresses (negative stress values) under tension.

The obtained Ogden material parameters are presented in Table 2 for all three sample types. Based on Drucker stability criteria, the material models are stable for all strain ranges as material parameters are positive. Interestingly, the obtained parameters differ significantly between the sample types, which indicates that the corresponding materials are not the same. We obtain the highest shear modulus for the porous samples and the lowest for the printed samples. The nonlinearity is slightly higher for the printed and porous samples than for the molded ones. We note that the porous samples still result in the lowest overall nominal stresses in Fig. 6. This can be attributed to the fact that these samples contain less material that can bear the load for the same volume due to the pores.

**Table 2:** Material parameters of the one-term Ogden model for molded, printed, and porous samples obtained from the inverse parameter identification approach.

Type	µ (kPa)	α
Molded	11.7	2
Printed	6.5	5.6
Porous	25.1	5.2

**Figure 7:**
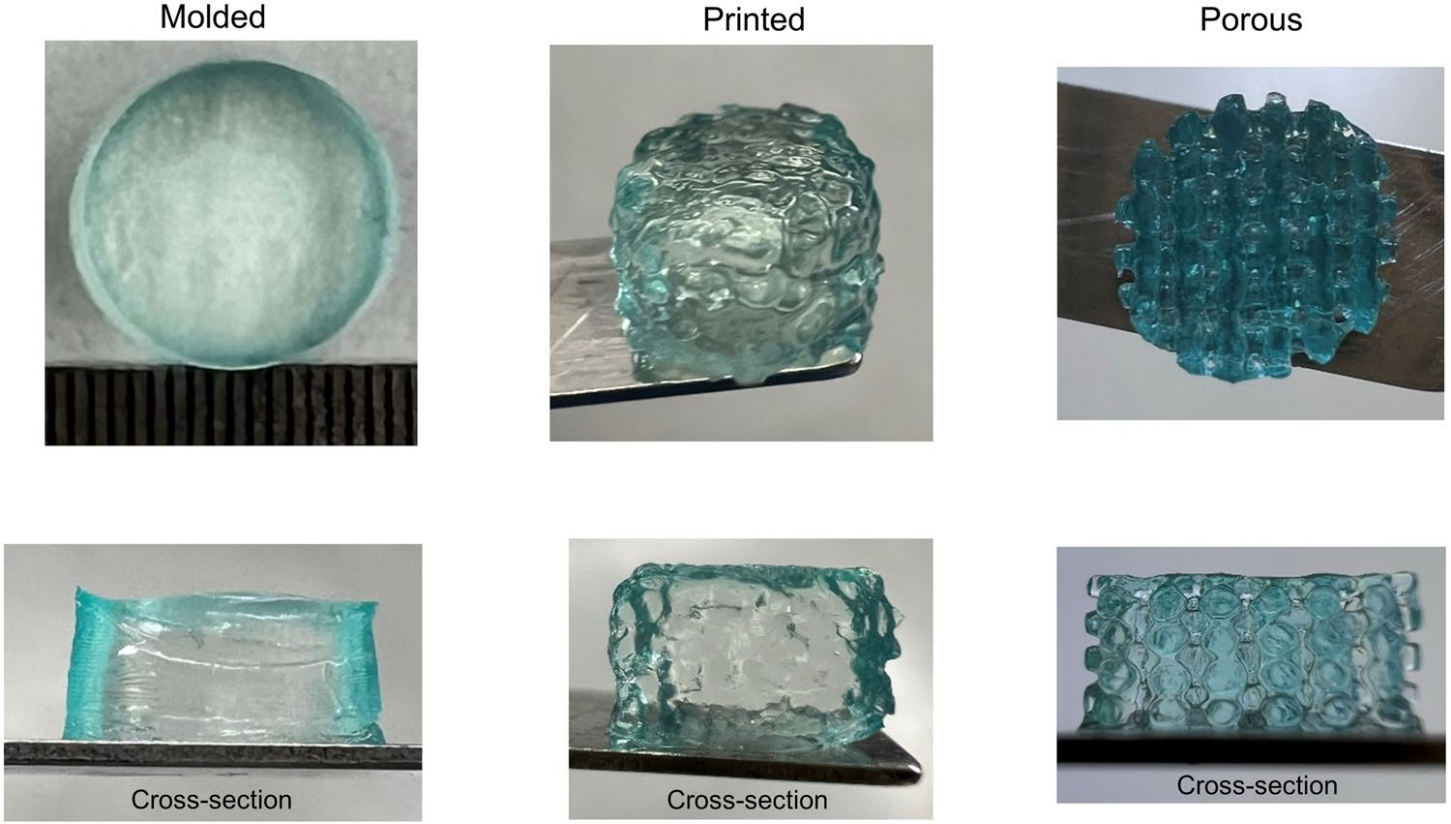
The effect of sample type on the crosslinking pattern. The overall and cross-sectional views of crosslinking patterns in molded, printed and porous samples are presented in top and bottom rows, respectively.

**Figure 8:**
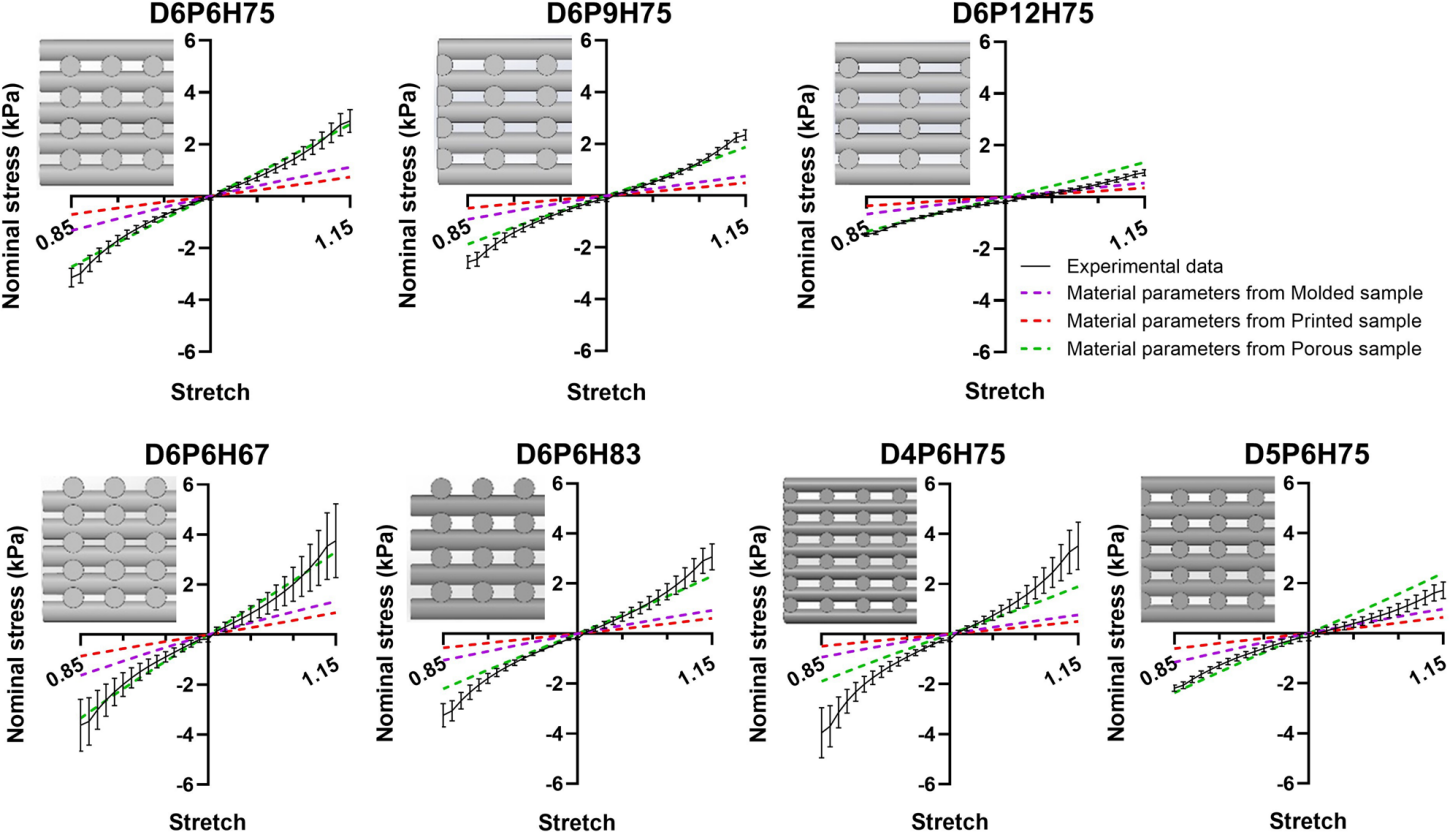
Comparison of nominal stresses during compression-tension loadings from experiments with predictions from FE simulations. FE simulations were performed using material parameters determined from molded, printed, and porous samples, respectively.

We attribute the observation that the shear moduli as a measure of the stiffness differed notably for the different types of samples (Table 2) to a difference in the layer bonding and crosslinking between molded, printed, and porous samples. As we observed that the specimen size could significantly affect the material properties after crosslinking (Fig. S1), we studied the effect of crosslinking on the porous and non-porous samples in more detail. We colored the 0.1 M CaCl_2_ using blue food color, crosslinked the three different sample types for ten minutes, and then washed with Hanks' Balanced Salt Solution (HBSS) to stop the reaction. As shown in Fig. 7, the blue color is mostly seen in the outer areas of molded and printed samples, indicating that the calcium ions could not penetrate the inner regions. In contrast, the blue color is seen in all areas of the porous sample confirming its different crosslinking patterns and high calcium ions penetration. Our observations agree with a previous study investigating the crosslinking pattern of AG hydrogels^43^. They showed that the diffusion of calcium ions is inhomogeneous and more pronounced in regions in direct contact with the hydrogel. The higher penetration and direct contact of internal surfaces with calcium chloride may result in more solidified regions in the porous samples than in non-porous samples and, therefore, higher material stiffness (Fig. 8). While the calcium diffusion could be different from that of the dye molecules, the observed results highlight the difference in the diffusion patterns between the three different samples. Also longer crosslinking times could have led to more homogeneous patterns, but to maintain the expose time to CaCl_2_ as short as possible as we plan to include cells in the future.

### FE model validation

After the calibration of material model parameters, we use the three identified parameter sets (molded, printed, and porous) for predicting the mesostructures' behavior in compression-tension loading. Figure 8 shows the results for the mesostructures mechanically tested in Ref.^21^. The simulations using molded and printed material parameters underestimate the experimentally recorded response of all seven mesostructures. In contrast, in most cases, the porous material parameters predict the mesostructures' mechanical behavior correctly, especially for compression loading. Still, there is a high deviation for the D4P6H75 sample. We attribute this to the fact that the actual printed filament diameter differed from the designed values, as described in detail in Ref.^21^. Unfortunately, as we had difficulties with printing and layer bonding, we were forced to increase the printing pressure, leading to higher effective printed filament diameters.

**Figure 9:**
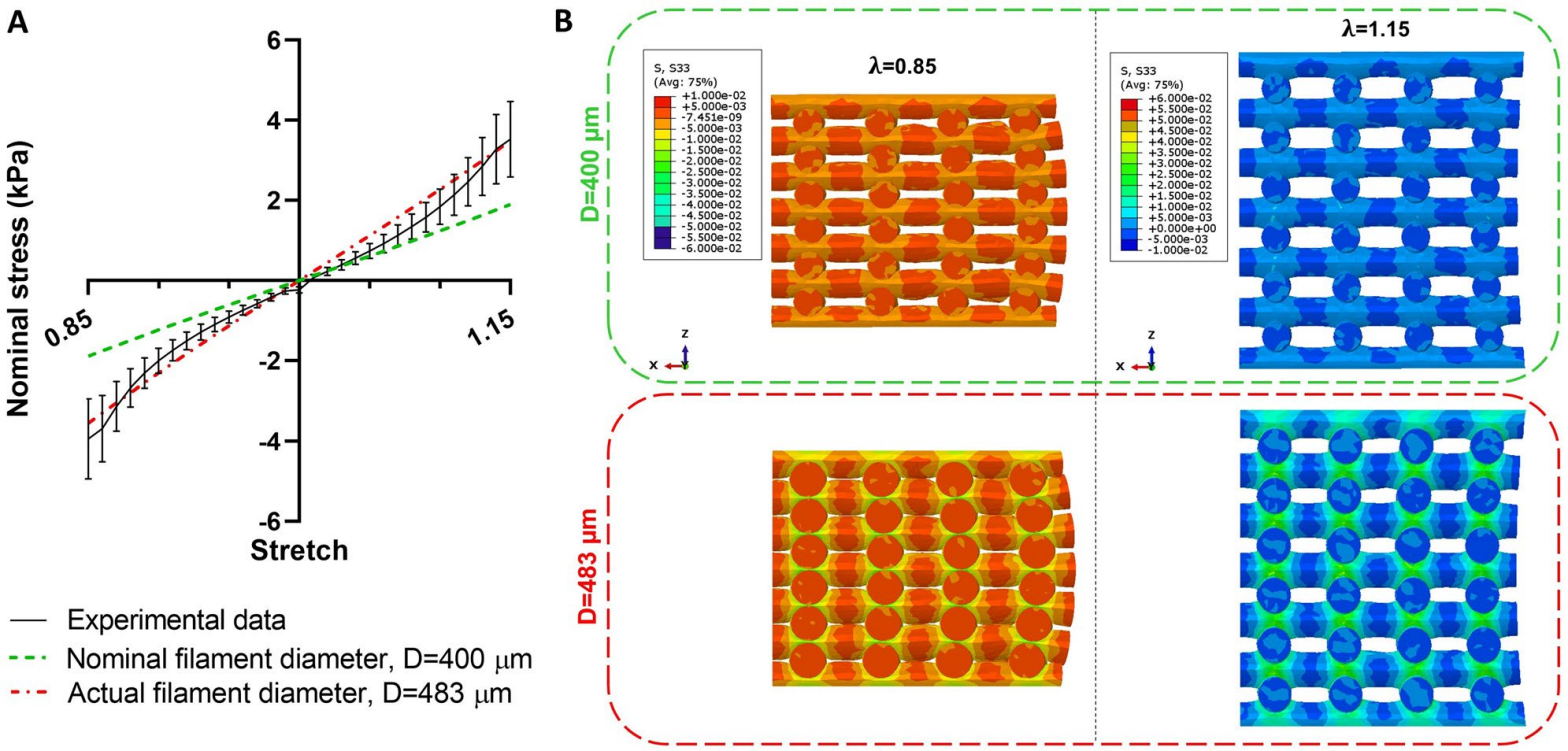
The importance of accounting for the actual filament diameter to allow for accurate numerical predictions for the mesostructure D4P6H75. (**A**) Nominal stress from numerical analyses versus experimental data. (**B**) The cross-sectional stress distribution at stretches of 0.85 and 1.15 are presented on the right.

Therefore, we also redesigned the FE model of this sample and changed the filament diameter from 400 to 483 µm (actual diameter measured for the experimental samples in Ref.^21^). Figure 9A demonstrates that when accounting for the actual diameter, also the model-predicted mechanical behavior agrees well with the experimentally recorded response. Figure 9B shows the corresponding cross-sectional stress distribution of the two types of D4P6H75 samples. It can be seen that by increasing the diameter from 400 to 483 µm, the maximum stress levels increase in both compression and tension.

### Prediction of mesostructures' mechanical properties

**Figure 10:**
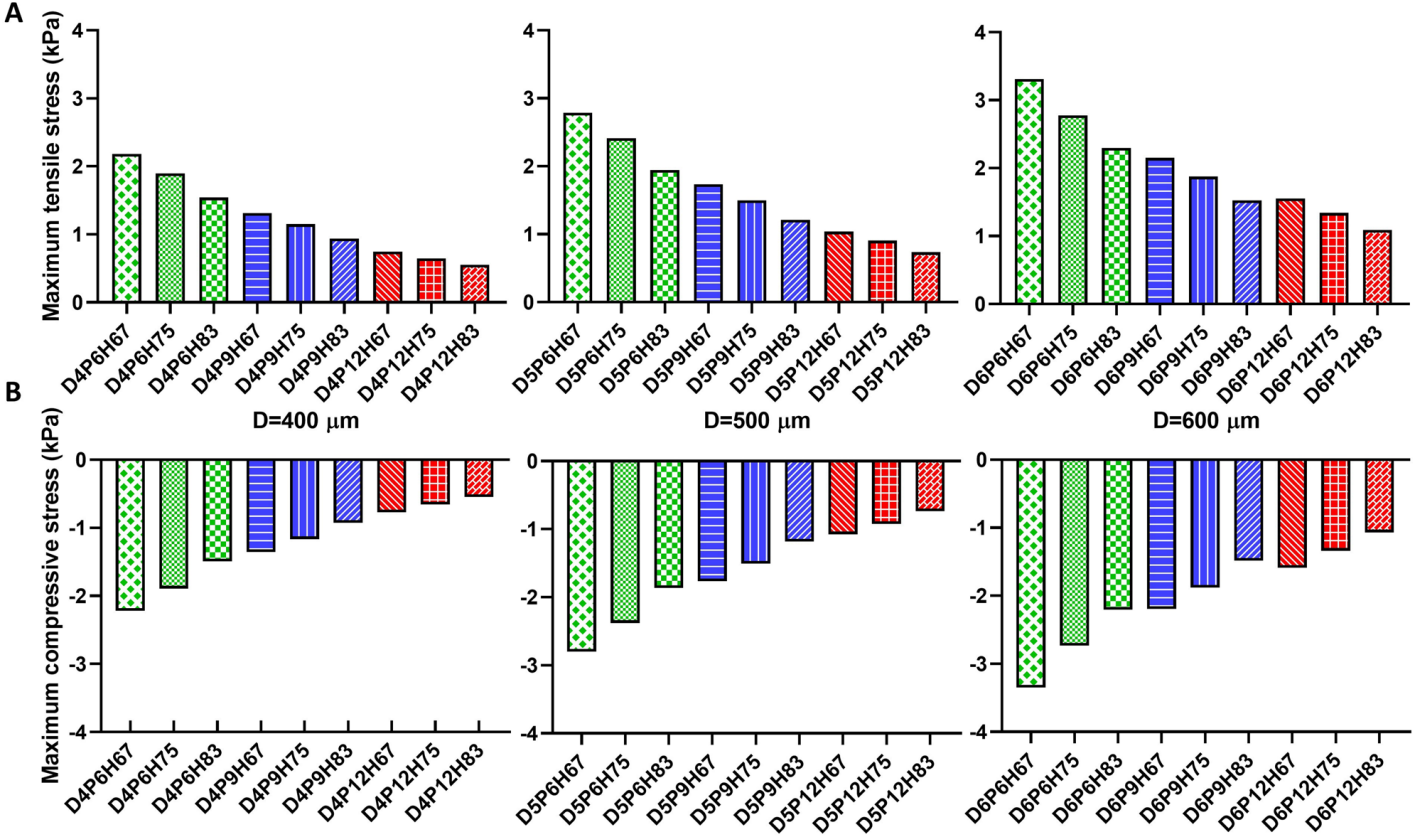
The effect of filament diameter, pore size and layer height on the maximum stresses for different mesostructures in tension (**A**) at stretch 1.15 and compression (**B**) at stretch 0.85. Their absolute values increase from left to right: for increasing filament diameter from 400 to 600 µm, increasing percentage of layer height to filament diameter from 67 to 83, and increasing pore size from 600 to 1200 µm.

After validating our FE approach, we analyzed other possible mesostructures with filament diameters of 400, 500, and 600 µm, pore sizes of 600, 900, and 1200 µm, and layer heights of 67, 75, and 83% of the filament diameter, which were not printed and mechanically tested in our previous study^21^. We evaluated the maximum stresses in compression and tension for stretches of 0.85 and 1.15, respectively, as presented in Fig. 10. It can be seen that by increasing the filament diameter while keeping pore size and the layer height to filament diameter ratio unchanged, the compressive and tensile stresses increase. We observe the same trend when decreasing pore size or the layer height to filament ratio. The different mesostructures cover a wide range of maximum stresses from 0.55 to 3.31 kPa in tension and -0.54 to -3.35 kPa in compression.

## Discussion

This study aimed to predict the mechanical behavior of bioprinted structures using finite element (FE) simulations to tune the properties to match the target tissue without the need for common trial-and-error experimental approaches^41,42^. Initially, we determined material model parameters for the matrix material, alginate-gelatine (AG, alginate 2% (w/v)-gelatin 5% (w/v)) hydrogels through an inverse approach based on FE simulations using the hyperelastic one-term Ogden material model and uniaxial compression-tension experimental data from our previous study^21^. By using the FE model, we could simulate the actual boundary conditions of the testing setup to ensure realistic predictions^31^.

We have identified material parameters based on average experimental data from porous samples. Although finding a single material model to fit both compression and tension data simultaneously can be challenging, we achieved good agreement between the model predictions and the experimental data (Fig. 6A). While the one-term Ogden model can represent the material behavior of AG bioinks in compression-tension loading reasonably well, using other hyperelastic models could be beneficial in the future to even better capture the experimentally observed nonlinearities.

After calibrating the material parameters, we predicted the remaining mesostructures' behavior using the three sets of material parameters. We observed that the FE model using the parameter set from porous samples could quite accurately predict the actual experimental response, while predictions based on parameters from molded and printed samples were completely off. This again highlights that the actual material properties of the matrix may be different for porous than for printed or molded samples, so that it is crucial to also use experimental data from porous samples for the identification of model parameters for FE simulations. We note, however, that this effect will highly depend on the particular material and crosslinking procedure used. Furthermore, we assumed homogeneous material properties throughout the specimen during the inverse parameter identification, which seems to be not fully valid for molded and printed samples regarding the crosslinking patterns observed in Fig. 7. This further supports that porous samples should be used to inversely characterize the material.

Using our validated FE model, we have finally performed simulations predicting the mechanical response for mesostructures that had not been previously tested experimentally. Increasing the filament diameter in mesostructures with similar pore size and layer height to filament diameter ratio led to increased maximum stresses (Fig. 10), similar to what had been observed in a previous study on 3D printed polylactic acid scaffolds^30^. This can be attributed to a higher amount of material to resist the loading within the same volume and increased bonding strength due to more layer penetrations for these samples. Moreover, decreasing the pore size without changing the filament diameter and layer height to filament ratio results in more material within the same volume and thus increased stress levels. Similarly, a lower layer height to filament ratio leads to higher layer penetration and bonding strength to resist the exerted deformation, resulting in higher stresses in agreement with the results of the previous study^30^. Our results in Fig. 10 show that by changing the mesostructure, the maximum stress can increase/decrease six times. Through the procedure presented in the current work, it is thus feasible to tune the structure's mechanical properties before any fabrication, e.g., to better match the properties of the target tissue. The properties of structures investigated here cover a wide range that is relevant for different soft tissues.

## Conclusion

In this study, we showed that finite element (FE) simulations can be used successfully for predicting and tuning the mechanical properties of 3D bioprinted hydrogel mesostructures, e.g., to match the properties of the target tissue. In addition, we have highlighted that the fabrication method and porosity significantly affect the material properties of alginate-gelatin hydrogels after crosslinking. As a result, when aiming to predict the mesostructures' mechanical properties through FE simulations, the material parameters need to be determined from experimental data of porous samples. Our study demonstrates the applicability of computational simulation models in hydrogel 3D bioprinting to enhance the progression speed in this field. In the future, the presented modeling and simulation approach can also be utilized for cell-laden hydrogel bioprinting to significantly decrease the number of experimental efforts required to reach the desired tissue construct.

### Supplementary Information

Supplementary Information.

## Data Availability

The datasets generated and/or analyzed during the current study are available from the corresponding author on reasonable request.
