# Supplementary material for: Predicting the hyperelastic properties of alginate-gelatin hydrogels and 3D bioprinted mesostructures
Source: Sci Rep. 2023 Dec 9;13:21858. doi: 10.1038/s41598-023-48711-3 (PMC10710406; doi:10.1038/s41598-023-48711-3)
Supplement: Supplementary file 1 — Supplementary Information. [file 41598_2023_48711_MOESM1_ESM.docx]

**Supplementary Information:**

**Predicting the hyperelastic properties of alginate-gelatin hydrogels and 3D bioprinted mesostructures**

**Anahita Ahmadi Soufivand, Silvia Budday***

Institute of Continuum Mechanics and Biomechanics, Department of Mechanical Engineering, Friedrich-Alexander-Universität Erlangen-Nürnberg, 91058 Erlangen, Germany.

* Corresponding author: silvia.budday@fau.de

We performed the following rheological tests to investigate how the sample dimension affects the mechanical properties of the alginate gelatin hydrogel after crosslinking. We tested two groups of crosslinked and control (uncrosslinked) hydrogels and used three heads for testing with diameters of 8, 20, and 40 mm. The test steps were:

1. Placing hydrogel on the bottom plate of the rheometer, lowering the upper head to maintain a 500 micrometers gap and making a thin film, and removing the extra hydrogel around the head using a spatula (The diameters of three groups of hydrogel samples were 8, 20, and 40 mm with a height of 0.5 mm.)
2. Cooling the hydrogel at 10°C for 5 minutes (Similar to the condition before crosslinking in making molded and printed samples)
3. Time sweep for 10 minutes:
   1. crosslinked group: adding CaCl_2_ to the hydrogel film right before starting
   2. control group: without crosslinking
4. Measuring and comparing storage modulus between crosslinked and control group


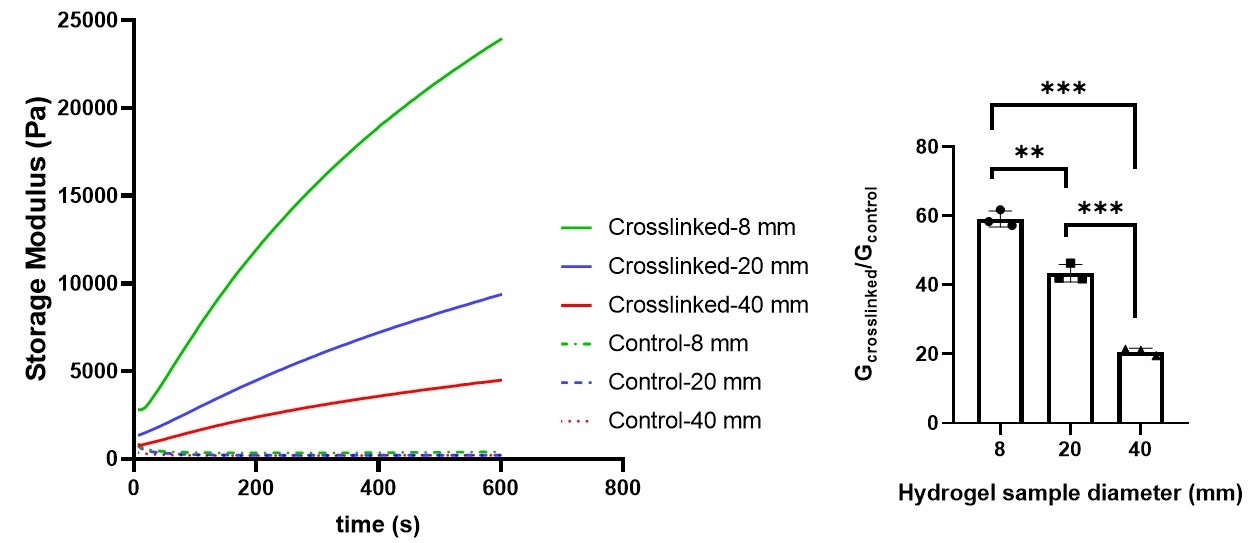


Figure S1. The effect of sample diameter on the mechanical properties of alginate gelatin hydrogels after crosslinking. The variation of storage modulus during 10 minutes of testing are shown on the left for crosslinked and control (uncrosslinked) groups. The storage modulus of crosslinked to the control group ratio is presented on the right, significance value **p < 0.01, ***p < 0.001.

From Figure S1, we can see that by increasing the hydrogel sample diameter, the storage modulus decreases significantly after crosslinking. This may be attributed to the lower penetration of calcium ions into the inner parts of the sample and more uncrosslinked regions, resulting in lower mechanical stiffness.
